# Naringenin Attenuates Methotrexate-Induced Nephrotoxicity Accompanied by Alterations in Oxidative Stress, Inflammatory, Apoptotic, and Endoplasmic Reticulum Stress Responses

**DOI:** 10.3390/ijms27135973

**Published:** 2026-07-03

**Authors:** Arzum Arzu, Zuhal Uckun Sahinogullari, Serife Efsun Antmen, Gokhan Nur, Safak Sandayuk

**Affiliations:** 1 Department of Pharmaceutical Toxicology, Institute of Health Sciences, Mersin University, Mersin 33343, Turkey; arzum.arzu1@outlook.com; 2 Department of Pharmaceutical Toxicology, Faculty of Pharmacy, Mersin University, Mersin 33169, Turkey; 3 Department of Biochemistry, Faculty of Pharmacy, Mersin University, Mersin 33169, Turkey; eantmen@mersin.edu.tr; 4 Department of Biomedical Engineering, Faculty of Engineering and Natural Sciences, Iskenderun Technical University, Hatay 31200, Turkey; gokhan.nur@iste.edu.tr; 5 Department of Molecular Biology, Faculty of Science and Arts, Kafkas University, Kars 36100, Turkey; safak.kars@hotmail.com

**Keywords:** methotrexate, nephrotoxicity, naringenin, oxidative stress, endoplasmic reticulum stress, apoptosis

## Abstract

Methotrexate (MTX) is widely used in the treatment of malignancies and inflammatory disorders, but nephrotoxicity remains a major adverse effect. Naringenin (NAR), a natural flavonoid, has antioxidant, anti-inflammatory, and nephroprotective properties. This study investigated the potential protective effects of NAR against MTX-induced nephrotoxicity at biochemical, molecular, and histopathological levels. Forty-two adult male Wistar albino rats were assigned to seven groups (n = 6): Control, CMC (carboxymethyl cellulose), NAR100, MTX, and MTX combined with NAR (25, 50, or 100 mg/kg/day). NAR was administered for 7 days, with MTX given on day 3. Renal function, histopathology, and genes associated with oxidative stress, apoptosis, endoplasmic reticulum stress, and inflammation were evaluated. MTX administration caused marked renal damage, increased creatinine and BUN levels, elevated apoptosis-, inflammation-, and ER stress-related gene expression, and suppressed antioxidant defense-related genes. However, 50 and 100 mg/kg/day NAR attenuated these alterations, with greater effects at 100 mg/kg/day. Histopathological damage was attenuated by NAR treatment, although complete recovery was not observed. These findings suggest that NAR may protect against MTX-induced nephrotoxicity through the modulation of pathways associated with oxidative stress, inflammation, apoptosis, and ER stress. However, the persistence of certain histopathological alterations indicates that structural recovery of renal tissue may take a longer period compared with molecular changes.

## 1. Introduction

The kidneys are particularly vulnerable to drug-induced toxicity due to their elevated metabolic activity and continuous exposure to circulating xenobiotics [1,2]. The nephrotoxicity associated with chemotherapeutic agents remains a major complication in cancer therapy, potentially causing treatment delays or interruptions, prolonged hospitalization, and increased mortality rates. Therefore, renal damage induced by anticancer agents is considered a significant factor limiting therapeutic efficacy [3].

Among these agents, methotrexate (MTX), a folic acid antagonist, is one of the most commonly used antineoplastic agents and is widely employed in the treatment of malignancies such as acute lymphoblastic leukemia, along with autoimmune and inflammatory disorders, including psoriasis and rheumatoid arthritis [4]. Nearly 90% of administered MTX is cleared through renal excretion, making the kidneys particularly vulnerable to MTX-induced toxicity. Despite its therapeutic efficacy, nephrotoxicity remains among the most clinically relevant toxicities associated with MTX administration. In addition to renal toxicity, MTX may also induce mucositis, hepatotoxicity, and gastrointestinal complications [5].

The mechanisms underlying MTX-induced nephrotoxicity are complex and multifactorial, and the precise molecular interactions involved in its pathogenesis have not yet been fully elucidated [6]. One of the primary mechanisms involves the accumulation of MTX and its major metabolite, 7-hydroxy-MTX, within renal tubules, a key contributor to MTX-induced kidney injury [7]. In addition, oxidative stress plays an important part in MTX-caused renal injury [8], and elevated generation of reactive oxygen species (ROS) contributes to the disruption of antioxidant defense systems [9]. This imbalance contributes to mitochondrial dysfunction and DNA damage, and ultimately activates apoptotic and inflammatory pathways that lead to renal injury [6,9].

Recent studies have also highlighted the involvement of endoplasmic reticulum (ER) stress and its close association with oxidative stress and apoptotic pathways in drug-induced renal injury [10]. Excessive ER stress may initiate unfolded protein response (UPR)-related signaling pathways through glucose-regulated protein 78 (GRP78)-mediated activation of protein kinase RNA-like endoplasmic reticulum kinase (PERK), inositol-requiring enzyme 1α (IRE1α), and activating transcription factor 6 (ATF-6) pathways [11]. Persistent ER stress may subsequently promote pro-apoptotic mediators like C/EBP homologous protein (CHOP) and caspase-12, thereby contributing to ER stress-mediated apoptosis [10]. However, the contribution of ER stress-associated signaling pathways in MTX-induced nephrotoxicity and their modulation by natural compounds remains insufficiently understood.

Over the past decade, heightened attention has been given to studies demonstrating that natural products with antioxidant properties may help alleviate the detrimental effects associated with anticancer agents. Plant-derived phenolic and flavonoid compounds have gained significant attention because of their diverse biological and pharmacological activities, particularly their antioxidant, anti-apoptotic, and anti-inflammatory activities [8]. Naringenin (4′,5,7-trihydroxyflavanone, NAR), a flavonoid and the aglycone form of naringin (NRG), is naturally found in various plants and dietary sources, especially citrus fruits [12,13]. NAR exhibits diverse biological activities that include potent antioxidant, nephroprotective, and anti-inflammatory properties [12,14].

Previous studies have demonstrated that NAR attenuates oxidative stress, enhances endogenous antioxidant enzyme activity, and alleviates histopathological damage in various experimental models [12,15,16]. Emerging evidence also suggests that NAR exerts beneficial effects through the modulation of multiple cellular signaling pathways involved in oxidative stress, inflammation, apoptosis, and ER stress-related responses [15]. Therefore, NAR may have an ameliorative effect against MTX-caused nephrotoxicity.

A literature review revealed that studies investigating the ameliorative effects of NRG against MTX-induced nephrotoxicity have been reported [5,17]. Although NRG is the glycoside form of NAR, differences in their biological activity and antioxidant properties have been reported [15,18]. To our knowledge, no studies have previously examined the nephroprotective effects of NAR in MTX-induced nephrotoxicity by evaluating oxidative stress, apoptosis-related genes, ER stress-associated signaling pathways, inflammatory responses, biochemical parameters, and histopathological alterations in renal tissues.

## 2. Results

### 2.1. Serum BUN and Creatinine Levels

**Figure 1:**
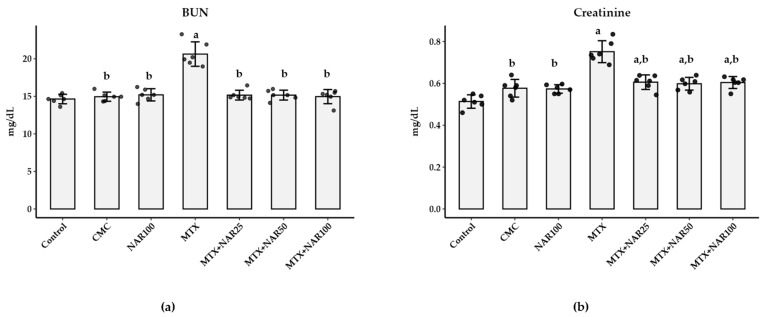
Effects of NAR on serum BUN (**a**) and creatinine (**b**) levels in the nephrotoxicity induced by MTX. Data are indicated as mean ± SD. ^a^ *p* < 0.05 vs. the control group; ^b^ *p* < 0.05 vs. the MTX group. Individual data points represent individual animals. CMC: carboxymethyl cellulose; NAR: naringenin; MTX: methotrexate; BUN: blood urea nitrogen.

Figure 1 summarizes the serum creatinine and BUN findings obtained from the control and experimental groups. Both serum creatinine and BUN levels were significantly elevated in the MTX group relative to the control group (*p* < 0.05). Similarly, the MTX group also had significantly more elevated serum creatinine and BUN levels than the CMC (carboxymethyl cellulose), NAR100, MTX+NAR25, MTX+NAR50, and MTX+NAR100 groups (*p* < 0.05). No statistically significant differences were detected among the MTX+NAR treatment groups with respect to either parameter (*p* > 0.05). However, serum creatinine levels in the MTX+NAR groups were significantly more elevated compared with those in the control group (*p* < 0.05). These outcomes demonstrate that NAR administration attenuated the elevations in serum BUN and creatinine levels caused by MTX treatment.

### 2.2. CAT and GPX mRNA Expression Levels

**Figure 2:**
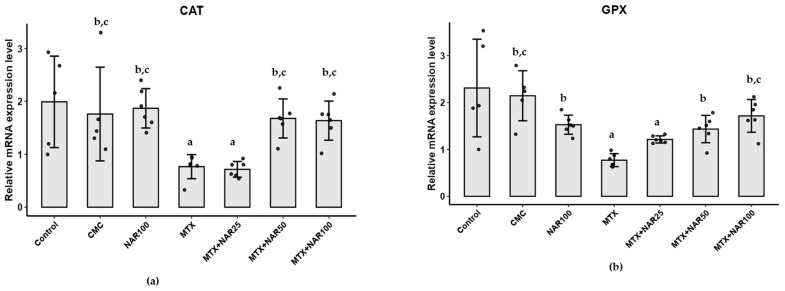
Effects of NAR on *CAT* (**a**) and *GPX* (**b**) mRNA expression levels in MTX-induced nephrotoxicity. Data are indicated as mean ± SD. ^a^ *p* < 0.05 vs. the control group; ^b^ *p* < 0.05 vs. the MTX group; ^c^ *p* < 0.05 vs. the MTX+NAR25 group. Individual data points represent individual animals. NAR: naringenin; MTX: methotrexate; *CAT*: catalase; *GPX*: glutathione peroxidase.

As displayed in Figure 2, *CAT* and *GPX* mRNA expression levels were significantly decreased in the MTX-treated group relative to the control, CMC, and NAR100 groups (*p* < 0.05). *CAT* and *GPX* mRNA expression levels did not differ significantly between the MTX and the MTX+NAR25 groups (*p* > 0.05). However, the expression levels of *CAT* and *GPX* were significantly increased in the MTX+NAR50 and MTX+NAR100 groups versus the MTX group (*p* < 0.05). In addition, *CAT* and *GPX* expression levels in the MTX+NAR100 group were significantly more elevated than those in the MTX+NAR25 group (*p* < 0.05). Also, *CAT* expression levels in the MTX+NAR50 group were significantly higher relative to those in the MTX+NAR25 group (*p* < 0.05). These outcomes indicate that moderate and high doses of NAR, particularly at higher doses, attenuated MTX-induced suppression of antioxidant defense-related gene expression.

### 2.3. NF-κB mRNA Expression Levels

**Figure 3:**
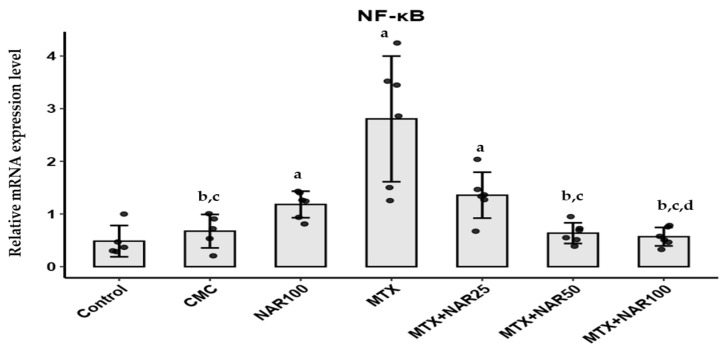
Effects of NAR on the expression levels of *NF-κB* mRNA in the nephrotoxicity induced by MTX. Data are indicated as mean ± SD. ^a^ *p* < 0.05 vs. the control group; ^b^ *p* < 0.05 vs. the MTX group; ^c^ *p* < 0.05 vs. the MTX+NAR25 group; ^d^ *p* < 0.05 vs. the NAR100 group. Individual data points represent individual animals. CMC: carboxymethyl cellulose; NAR: naringenin; MTX: methotrexate; *NF-κB*: Nuclear factor kappa B.

As illustrated in Figure 3, the expression levels of *NF-κB* mRNA were significantly elevated in the MTX group relative to the control and CMC groups (*p* < 0.05). A similar pattern was observed in the MTX+NAR25 group, which also displayed higher expression levels than the control and CMC groups (*p* < 0.05). The expression levels of *NF-κB* mRNA did not differ significantly among the NAR100, MTX, and MTX+NAR25 groups (*p* > 0.05). However, the expression levels of *NF-κB* in the MTX+NAR50 and MTX+NAR100 groups were significantly reduced relative to those in the MTX and MTX+NAR25 groups (*p* < 0.05). In addition, *NF-κB* expression levels were determined to be statistically decreased in the MTX+NAR100 group versus the NAR100 group. These outcomes suggest that the moderate and high doses of NAR attenuated MTX-induced inflammation (Figure 3).

### 2.4. UPR-Related mRNA Expression Levels

**Figure 4:**
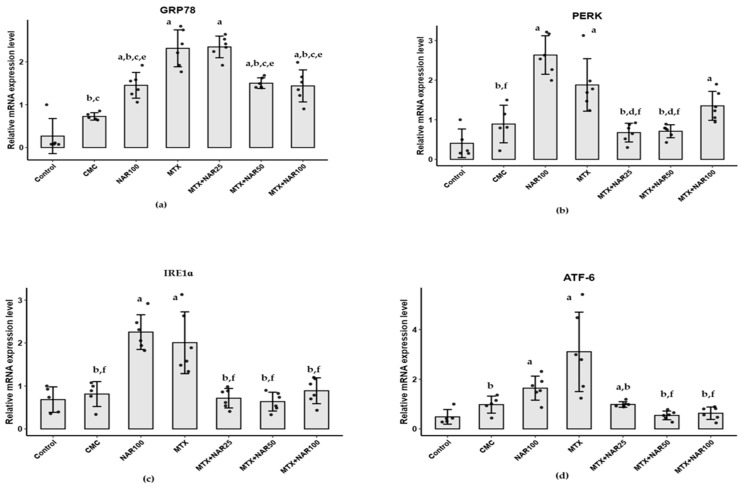
Effects of NAR on *GRP78* (**a**), *PERK* (**b**), *IRE1α* (**c**), and *ATF-6* (**d**) mRNA expression levels in MTX-induced nephrotoxicity. Values are presented as mean ± SD. ^a^ *p* < 0.05 vs. the control group; ^b^ *p* < 0.05 vs. the MTX group; ^c^ *p* < 0.05 vs. the MTX+NAR25 group; ^d^ *p* < 0.05 vs. the MTX+NAR100 group, ^e^ *p* < 0.05 vs. the CMC group, ^f^ *p* < 0.05 vs. the NAR100 group. Individual data points represent individual animals. CMC: carboxymethyl cellulose; NAR: naringenin; MTX: methotrexate. *GRP78*: Glucose-regulated protein 78; *PERK*: Protein kinase RNA-like endoplasmic reticulum kinase; *IRE1α*: Inositol-requiring enzyme 1α; *ATF-6*: Activating transcription factor 6.

The MTX group exhibited a substantial increase in the mRNA expression levels of *GRP78*, *ATF-6*, *IRE1α,* and *PERK* relative to both the control and CMC groups (*p* < 0.05). Furthermore, the NAR100 group demonstrated significantly elevated transcript levels of *GRP78*, *IRE1α*, and *PERK* relative to the control and CMC groups (*p* < 0.05). Regarding *GRP78*, its expression in the MTX and MTX+NAR25 groups was markedly higher than that observed in the CMC, NAR100, MTX+NAR50, and MTX+NAR100 groups (*p* < 0.05). In addition, the expression levels of *GRP78* in the MTX+NAR50 and MTX+NAR100 groups remained significantly higher versus the control and CMC groups (*p* < 0.05). As for *PERK*, the MTX and NAR100 groups demonstrated significantly higher expression levels than the MTX+NAR25 and MTX+NAR50 groups (*p* < 0.05). However, no statistically meaningful variations were determined among the NAR100, MTX, and MTX+NAR100 groups (*p* > 0.05). For *IRE1α*, the expression levels in the MTX and NAR100 groups were significantly greater than those in the MTX+NAR25, MTX+NAR50, and MTX+NAR100 groups (*p* < 0.05). In a similar manner, *ATF-6* expression in the MTX group was significantly more elevated than in the MTX+NAR25, MTX+NAR50, and MTX+NAR100 groups (*p* < 0.05). Furthermore, *ATF-6* expression levels in the NAR100 group were substantially higher relative to those in the MTX+NAR50 and MTX+NAR100 groups (*p* < 0.05). Overall, these findings suggest that NAR ameliorated MTX-induced ER stress and UPR-related signaling pathways, particularly at moderate and high doses (Figure 4).

### 2.5. ER Stress-Related Apoptotic mRNA Expression Levels

**Figure 5:**
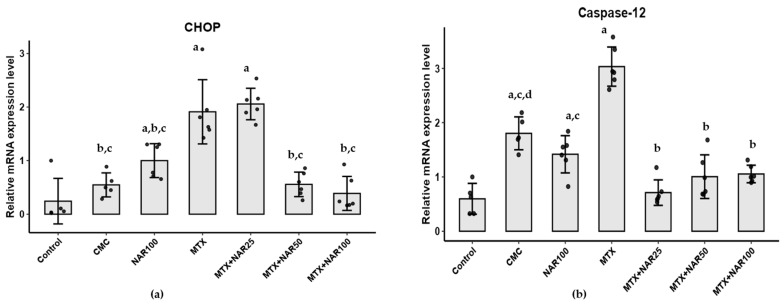
Effects of NAR on *CHOP* (**a**) and *Caspase-12* (**b**) mRNA expression levels in MTX-induced nephrotoxicity. Data are indicated as mean ± SD. ^a^ *p* < 0.05 vs. the control group; ^b^ *p* < 0.05 vs. the MTX group; ^c^ *p* < 0.05 vs. the MTX+NAR25 group; ^d^ *p* < 0.05 vs. the MTX+NAR50 group. Individual data points represent individual animals. CMC: carboxymethyl cellulose; NAR: naringenin; MTX: methotrexate; *CHOP*: C/EBP homologous protein.

The NAR100, MTX, and MTX+NAR25 groups exhibited significantly elevated *CHOP* mRNA transcript levels relative to the control group (*p* < 0.05). However, no statistically meaningful variations were detected among the NAR100, MTX, and MTX+NAR25 groups (*p* > 0.05). On the other hand, *CHOP* expression levels in the MTX+NAR50 and MTX+NAR100 groups were markedly lower than those observed in the MTX and MTX+NAR25 groups (*p* < 0.05). Regarding *caspase-12*, its expression was significantly higher in the CMC, NAR100, and MTX groups when compared with the control group (*p* < 0.05). Furthermore, *caspase-12* expression in the MTX group was determined to be substantially greater than in the MTX+NAR25, MTX+NAR50, and MTX+NAR100 groups (*p* < 0.05). There were no statistically significant differences identified among the MTX+NAR25, MTX+NAR50, and MTX+NAR100 groups (*p* > 0.05). In addition, prominent differences in *caspase-12* expression were noted between the MTX+NAR25 group and both the CMC and NAR100 groups, as well as between the MTX+NAR50 and CMC groups. Overall, these results suggest that the NAR ameliorated MTX-induced ER stress-mediated apoptotic signaling, particularly at moderate and high doses (Figure 5).

### 2.6. Bax and Bcl-2 mRNA Expression Levels

**Figure 6:**
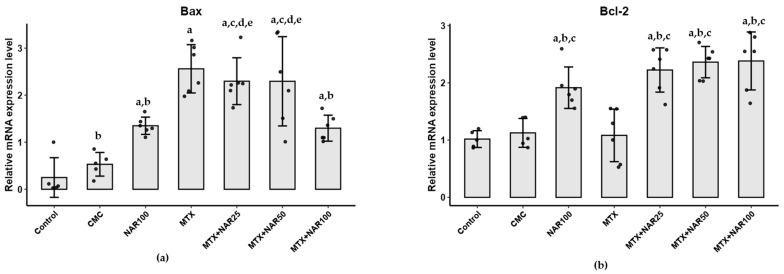
Effects of NAR on *Bax* (**a**) and *Bcl-2* (**b**) mRNA expression levels in the nephrotoxicity induced by MTX. Data are indicated as mean ± SD. ^a^ *p* < 0.05 vs. the control group; ^b^ *p* < 0.05 vs. the MTX group; ^c^ *p* < 0.05 vs. the CMC group; ^d^ *p* < 0.05 vs. the NAR100 group; ^e^ *p* < 0.05 vs. the MTX+NAR100 group. Individual data points represent individual animals. CMC: carboxymethyl cellulose; NAR: naringenin; MTX: methotrexate.

Compared to the control group, significantly elevated *Bax* mRNA expression levels were observed in the NAR100, MTX, MTX+NAR25, MTX+NAR50, and MTX+NAR100 groups (*p* < 0.05). In addition, *Bax* expression levels in the MTX, MTX+NAR25, and MTX+NAR50 groups were markedly higher than those in the CMC and NAR100 groups (*p* < 0.05). Conversely, the MTX+NAR100 group exhibited a substantial decrease in *Bax* mRNA expression relative to the MTX, MTX+NAR25, and MTX+NAR50 groups (*p* < 0.05). These results indicate that the administration of high-dose NAR may suppress the MTX-induced increase in *Bax* mRNA expression levels. *Bcl-2* mRNA expression levels were significantly higher in the NAR100, MTX+NAR25, MTX+NAR50, and MTX+NAR100 groups versus the control and CMC groups (*p* < 0.05). Conversely, *Bcl-2* expression levels in the MTX group were significantly lower than those in the NAR100, MTX+NAR25, MTX+NAR50, and MTX+NAR100 groups (*p* < 0.05). These results suggest that NAR administration increased *Bcl-2* expression levels in MTX-induced renal damage (Figure 6).

### 2.7. Caspase-3, Caspase-8, and Caspase-9 mRNA Expression Levels

**Figure 7:**
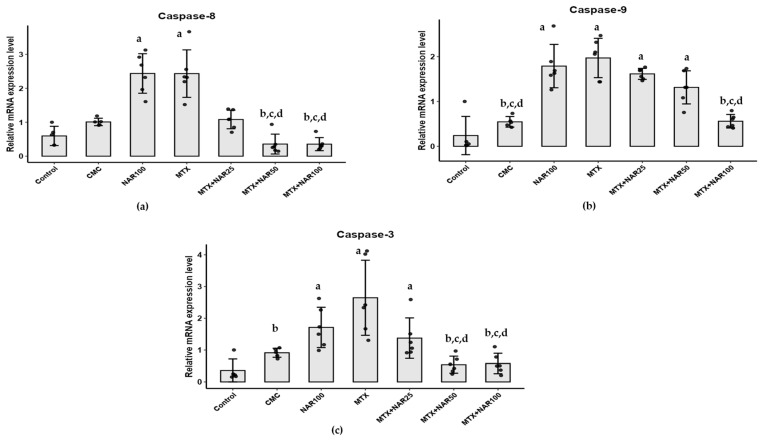
Effects of NAR on *Caspase-8* (**a**), *Caspase-9* (**b**), and *Caspase-3* (**c**) mRNA expression levels in MTX-induced nephrotoxicity. Data are indicated as mean ± SD. ^a^ *p* < 0.05 vs. the control group; ^b^ *p* < 0.05 vs. the MTX group; ^c^ *p* < 0.05 vs. the MTX+NAR25 group; ^d^ *p* < 0.05 vs. the NAR100 group. Individual data points represent individual animals. CMC: carboxymethyl cellulose; NAR: naringenin; MTX: methotrexate.

The mRNA expression levels of *caspase-3*, *caspase-8*, and *caspase-9* are presented in Figure 7.

The NAR100, MTX, and MTX+NAR25 groups demonstrated significantly elevated mRNA expression levels of *caspase-3*, *caspase-8*, and *caspase-9* relative to the control group (*p* < 0.05). Conversely, no statistically meaningful variations were detected among the NAR100, MTX, and MTX+NAR25 groups (*p* > 0.05). Regarding *caspase-3* and *caspase-8*, the MTX+NAR50 and MTX+NAR100 groups exhibited markedly reduced expression levels relative to the NAR100, MTX, and MTX+NAR25 groups (*p* < 0.05). As for *caspase-9*, a substantial decrease in expression levels was observed in the MTX+NAR100 group compared to the NAR100, MTX, and MTX+NAR25 groups (*p* < 0.05). These outcomes suggest that NAR may reduce MTX-induced caspase expression levels, with the most pronounced effect observed at higher doses.

### 2.8. Histopathological Findings

**Figure 8:**
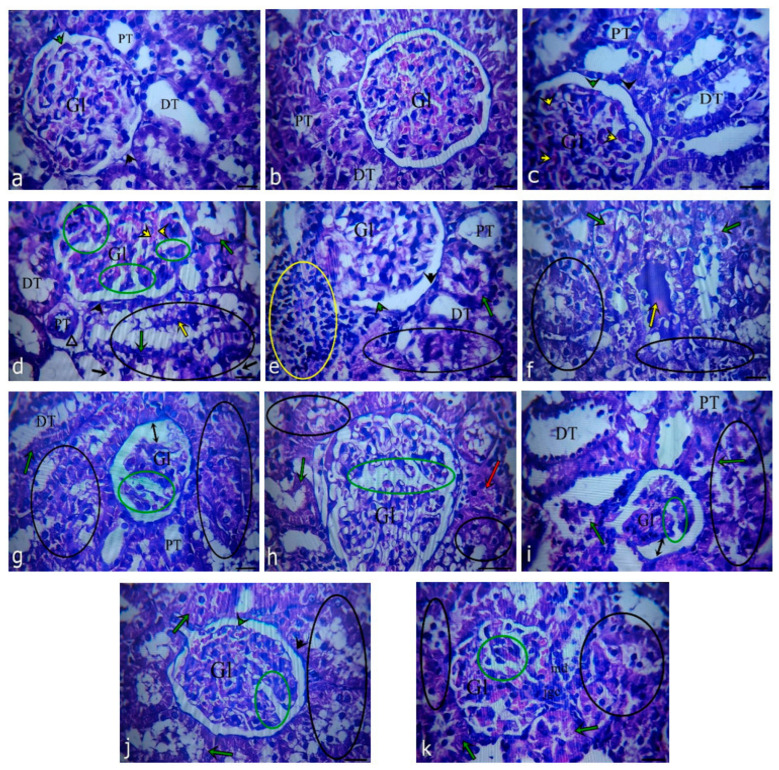
Representative photomicrographs of renal sections stained with hematoxylin and eosin (H&E) (Scale bar = 20 µm). (**a**) Control, (**b**) CMC, (**c**) NAR100, (**d**,**e**) MTX, (**f**,**g**) MTX+NAR25, (**h**,**i**) MTX+NAR50, and (**j**,**k**) MTX+NAR100 groups. GL: glomeruli; PT: proximal tubule; DT: distal tubule; parietal layer of Bowman’s capsule (black arrowhead); podocytes/visceral layer of Bowman’s capsule (green arrowhead); glomerular capillary tuft (yellow arrowhead); diffuse tubular degeneration (black circle); glomerular lobulation (green circle); intratubular cast (yellow arrow); degenerated tubular epithelial cells (green arrow); detachment from the basement membrane (triangle); pyknotic nuclei (black arrow); inflammatory cell infiltration (yellow circle); dilation of Bowman’s capsule and glomerular atrophy (double arrowhead); tubular necrosis (red arrow); macula densa (md); juxtaglomerular cells (jgc). H&E.

Histopathological findings are presented in Table 1 and Figure 8.

In the present study, kidney tissues were histopathologically evaluated for cortical congestion, glomerular atrophy, inflammatory cell infiltration, glomerular lobulation, tubular degeneration, and enlargement of Bowman’s capsule. No histopathological changes were observed in the control, CMC, and NAR100 groups, indicating normal renal histology. In contrast, the MTX group exhibited marked renal histopathological lesions, including the aforementioned lesions. Similar lesions were observed in the MTX+NAR25 group as well. However, the frequency and severity of these histopathological alterations were diminished in the MTX+NAR50 and MTX+NAR100 groups in comparison with the MTX and MTX+NAR25 groups. The results suggest that NAR administration at 50 and 100 mg/kg/day reduced MTX-associated renal histopathological damage.

## 3. Discussion

To our knowledge, the current report is the first study to document the ameliorative impacts of NAR against the nephrotoxicity induced by MTX in a rat model. The possible attenuating properties of NAR were examined by assessing parameters associated with oxidative stress-related markers, the inflammatory response, apoptosis-associated gene expression, ER stress-mediated signaling pathways, and histopathological alterations.

GPX and CAT serve as crucial antioxidant enzymes responsible for sustaining cellular redox homeostasis [19]. Prior literature has documented that MTX-induced nephrotoxicity models are characterized by elevated oxidative stress alongside a compromised antioxidant defense mechanism [5,20]. Similarly, in the current investigation, MTX administration significantly reduced the expression levels of *GPX* and *CAT* mRNA in renal tissue. Conversely, NAR administration significantly attenuated this reduction at doses of 50 and 100 mg/kg/day, with a more pronounced influence observed at the 100 mg/kg/day dose. This evidence suggests that NAR may contribute to the maintenance of cellular redox balance and a reduction in oxidative stress through its antioxidant properties.

Oxidative stress and inflammatory cytokines are closely interconnected and play important roles in acute renal injury caused by toxic agents [21]. Increased oxidative stress may stimulate the production of pro-inflammatory cytokines, whereas inflammatory mediators can further aggravate oxidative damage [21,22]. NF-κB is one of the most important regulatory pathways related to oxidative stress and inflammatory response, playing a central role in inflammatory processes [21,23]. Activation of the NF-κB pathway and its association with renal inflammation has previously been reported in experimental nephrotoxicity models induced by MTX [22,24]. In the current investigation, it was determined that MTX administration raised *NF-κB* gene expression in kidney tissue, while NAR administration reduced this increase, especially at doses of 50 and 100 mg/kg. Previous studies have reported that the anti-inflammatory influences of NAR may be related to the modulation of the NF-κB signaling pathway and the suppression of pro-inflammatory cytokine expression [23].

In the present study, MTX administration activated the three major UPR branches, namely *PERK*, *ATF-6*, and *IRE1α* in renal tissue. GRP78, located in the ER lumen, is considered the central regulator of the UPR [25]. Under ER stress conditions, GRP78 dissociates from ER stress sensor proteins, comprising PERK, ATF-6, and IRE1α, thereby activating these signaling pathways [11]. Prior literature has documented that the adaptive ER stress response initiated by an increase in GRP78 can progress to a pro-apoptotic process with a marked elevation in CHOP expression in prolonged ER stress [11,26]. CHOP is recognized as an important ER stress-associated pro-apoptotic transcription factor that promotes cell death when the adaptive UPR becomes insufficient [27]. CHOP activation can promote caspase-12 activation and the subsequent caspase cascade, ultimately inducing ER stress-mediated apoptosis [10]. In the current investigation, MTX administration increased the expression levels of *GRP78*, *ATF-6*, *IRE1α*, *PERK*, *CHOP*, and *caspase-12*, indicating activation of ER stress-associated pathways. In contrast, NAR treatment, particularly at moderate (50 mg/kg/day) and high doses (100 mg/kg/day), reduced the expression levels of these parameters, suggesting that NAR may exert protective effects against MTX-induced ER stress and ER stress-mediated apoptotic signaling. This effect is predicted to be due to NAR’s antioxidant properties reducing the cellular stress load and limiting the transition of the adaptive UPR to the pro-apoptotic phase. However, the mild increases observed in some parameters in the NAR100 group may be related to the dose-dependent effects of flavonoids on cellular redox homeostasis [28]. Importantly, these transcriptional changes were not accompanied by alterations in renal function parameters or histopathological injury, suggesting that they do not reflect overt renal toxicity. Previous studies have suggested that certain phytochemicals may activate adaptive cellular stress-response pathways, a phenomenon associated with hormesis [29,30]. Therefore, the modest increases observed in some ER stress-related markers in the NAR100 group may represent an adaptive cellular response rather than pathological ER stress. In contrast, under MTX-induced cellular stress conditions, the antioxidant and cytoprotective properties of NAR may attenuate excessive ER stress activation and contribute to the restoration of cellular homeostasis.

During the apoptotic process, the initiator caspases, namely caspase-8 (extrinsic pathway) and caspase-9 (mitochondrial intrinsic pathway) are activated, leading to activation of the effector caspase-3, which represents the final common step of both apoptotic pathways [12]. It has been reported that CHOP has the ability to trigger cell death via both the extrinsic apoptosis pathway (death receptor pathway) and the intrinsic apoptosis pathway if ER stress (UPR) is not resolved and persists for an extended period [10]. Furthermore, CHOP can induce apoptosis in cells through several molecular pathways, including the suppression of the anti-apoptotic protein Bcl-2 and the activation of caspase-12 [31,32]. Caspase-12, which functions as an ER-resident caspase, is selectively activated under ER stress conditions, leading to activation of caspase-9 and caspase-3 and ultimately initiation of apoptosis. Furthermore, CHOP can also contribute to the activation of caspase-8 involved in the extrinsic apoptotic pathway, thereby promoting cell death [10]. In the present study, MTX administration was found to significantly increase the expression levels of *caspase-3*, *caspase-8*, and *caspase-9* genes in kidney tissue. In contrast, NAR treatment significantly attenuated these increases, particularly at doses of 50 and 100 mg/kg.

CHOP is recognized as an important transcription factor that regulates the expression of BCL-2 family proteins, including Bax and Bcl-2, during apoptosis. Previous studies have shown that CHOP increases pro-apoptotic *Bax* expression while suppressing anti-apoptotic *Bcl-2* expression [10]. In the present study, MTX administration increased *Bax* gene expression and decreased *Bcl-2* gene expression in kidney tissue, whereas NAR treatment reversed these alterations by modulating the *Bax/Bcl-2* balance toward an anti-apoptotic direction. These findings suggest that NAR may exert protective effects on the mitochondrial apoptotic pathway.

The present data are generally consistent with previous studies reporting nephro-protective effects of other flavonoids. For example, hesperidin has been shown to attenuate nephrotoxicity through the modulation of NF-κB/TNF-α/IL-1β, Bax/Bcl-2, and ATF-6/IRE1α/PERK/GRP78 signaling pathways [26]. Similarly, chrysin [33] and nobiletin [25] have been reported to exert nephroprotective effects through regulation of oxidative stress, inflammation, apoptosis, and ER stress-associated pathways. In the present study, NAR likewise reduced the expression of *NF-κB*, *Bax*, *caspase-3*, *caspase-8*, *caspase-9*, *caspase-12*, *GRP78*, *PERK*, *IRE1α*, *ATF-6*, and *CHOP*, while increasing *Bcl-2* expression. Thus, the nephroprotective effects of NAR appear to share several mechanistic features with those reported for other flavonoids, particularly with respect to the regulation of pathways related to oxidative stress, inflammation, apoptosis, and ER stress. Collectively, these findings suggest that coordinated modulation of these interconnected cellular stress responses may represent common mechanisms underlying flavonoid-mediated nephroprotection.

Serum BUN and creatinine levels are widely used as biochemical indicators to evaluate renal dysfunction and kidney injury [33]. It has previously been reported that MTX administration leads to increased BUN and creatinine levels in experimental nephrotoxicity models [7,34]. Consistent with these reports, the present study demonstrated that MTX administration significantly increased serum BUN and creatinine levels, indicating MTX-induced renal dysfunction. In contrast, significant decreases in serum BUN and creatinine levels were observed in the NAR-treated groups compared to the MTX group. However, creatinine levels remained higher than in the control group and certain histopathological lesions persisted. These findings suggest that structural recovery of kidney tissue may require a longer time than functional recovery. This outcome is also supported by the histopathological findings of this investigation.

Histopathological examinations in this study revealed that MTX administration caused significant structural damage in renal tissue, characterized by cortical congestion, glomerular atrophy, inflammatory cell infiltration, glomerular lobulation, tubular degeneration, and enlargement of Bowman’s capsule. Similar histopathological alterations have previously been reported in experimental nephrotoxicity models induced by MTX [4,35]. In this investigation, the renal histopathological damage observed in the MTX group was similarly observed in the MTX+NAR25 group. Conversely, a decrease in the severity of histopathological damage was observed in the MTX+NAR50 and especially the MTX+NAR100 groups, but complete histopathological recovery was not determined. It has previously been reported that NAR can provide histopathological improvement in renal and other tissue damage caused by various toxic agents [14,36]. These findings are consistent with the results of the current investigation.

In our previous study on vancomycin (VCM)-induced nephrotoxicity, lower doses of NAR (25 and 50 mg/kg/day, especially at 25 mg/kg/day) showed more pronounced protective effects compared with the 100 mg/kg/day dose [12]. In contrast, the present MTX-induced nephrotoxicity model demonstrated more evident protective effects at 50 and 100 mg/kg/day doses, and especially at 100 mg/kg/day. These differences may be related to variations in the experimental models, nephrotoxic agents, and molecular pathways involved in VCM- and MTX-induced renal injury. Together, these outcomes suggest that the protective efficacy of NAR may be model- and dose-dependent. The basis of these differing dose–response patterns remains unclear. However, VCM and MTX exhibit distinct pharmacological and renal handling characteristics [37,38], which may influence renal exposure and the nature of nephrotoxic injury. Consequently, differences in the cellular responses triggered by these agents may contribute to variations in the optimal protective dose of NAR between experimental models. Nevertheless, as pharmacokinetic parameters were not evaluated in this investigation, this interpretation remains speculative and warrants further investigation.

This study has several limitations. First, the molecular pathways investigated were evaluated only at the mRNA expression level, and protein expression or activity analyses were not performed. Therefore, future studies incorporating Western blotting and immunohistochemical analyses are needed to confirm the present findings at the protein level. Second, histopathological evaluations were conducted at a single experimental time point using a semi-quantitative scoring approach, limiting the evaluation of the temporal progression of renal injury and recovery. Nevertheless, the histopathological findings were consistent with serum creatinine, BUN, and molecular marker results. Furthermore, although different doses of NAR were evaluated, different treatment durations were not investigated. Future studies involving multiple time points, quantitative histopathological analyses, and longer treatment periods may provide a more comprehensive understanding of the nephroprotective effects of NAR. Finally, well-designed clinical studies are required to translate these experimental findings into clinical practice.

## 4. Materials and Methods

### 4.1. Chemicals and Reagents

MTX (Kocsel firm, Istanbul, Turkey) and NAR were obtained from Sigma-Aldrich (St. Louis, MO, USA; Cat. No. N5893). All other chemicals and solutions utilized in this study were of the highest analytical grade and procured from standard commercial vendors.

### 4.2. Experimental Animals and Experimental Design

Ethical approval for the animal protocols was granted by the Mersin University Animal Experimentation Local Ethics Committee (Approval No: 2024/15), Turkey. The present study was carried out in strict compliance with current national and international guidelines for the care and welfare of laboratory animals.

A cohort comprising 42 adult male Wistar albino rats, weighing between 200 and 300 g, was utilized for the experiments. The subjects were maintained in a controlled laboratory environment with a regulated temperature of 25 ± 2 °C, a relative humidity of 55 ± 8% and a 12 h light/dark cycle, while enjoying ad libitum access to standard chow and water. Rats were randomly divided into seven groups (n = 6 per group):

1. Control group: Rats were administered a single dose of 0.9% NaCl intraperitoneally (i.p.) on day 3 of the experiment period.
2. CMC group: Rats were administered 0.5% carboxymethyl cellulose orally (CMC, p.o.) once daily for 7 days.
3. NAR100 group: NAR, suspended in CMC 0.5%, was administered orally at a dose of 100 mg/kg once daily for 7 days.
4. MTX group: Rats received a single intraperitoneal (i.p.) dose of 20 mg/kg of MTX on day 3.
5. MTX+NAR25, 6. MTX+NAR50, and 7. MTX+NAR100 groups: Rats were administered NAR orally at doses of 25, 50, and 100 mg/kg/day, respectively, once daily for 7 days. Also, a single intraperitoneal dose of MTX (20 mg/kg) was administered on day 3, one hour after NAR treatment, for each group.

During the experimental period, all chemicals were freshly prepared each day. The doses of NAR [12] and MTX [39] used in the present investigation were selected based on prior studies.

### 4.3. Sample Collection

Twenty-four hours after the final treatment, anesthesia was induced in the rats using a ketamine/xylazine combination, followed by euthanasia via cervical dislocation. For serum isolation, blood was drawn from the inferior vena cava and subjected to centrifugation at 4000× *g* for 10 min at 4 °C to obtain serum.

Kidney tissues were excised; right kidneys were stored at −80 °C for biochemical and molecular analyses, whereas left kidneys were fixed in 10% buffered formalin for histopathological examination.

### 4.4. Biochemical Analysis

The levels of serum creatinine and blood urea nitrogen (BUN) were quantified utilizing colorimetric kits (Otto Scientific, Ankara, Turkey; Cat. No. OttoBC139 and OttoBC157, respectively) by following the protocols provided by the manufacturer. The obtained values were presented in mg/dL.

### 4.5. mRNA Expression Analysis

Isolation of total RNA from renal tissue samples was performed employing the innuPREEP RNA Kit 2.0 (845-CS-1010250, IST, Berlin, Germany). Subsequently, complementary DNA (cDNA) synthesis was executed from the extracted RNA templates utilizing a High-Capacity cDNA Reverse Transcription Kit (Thermo Fisher Scientific, Waltham, MA, USA). To evaluate the transcriptional changes in *CAT*, *GPX*, *NF-κB*, *Bax*, *Bcl-2*, *caspase-3*, *caspase-8*, *caspase-9*, *caspase-12*, *GRP78*, *ATF-6*, *PERK*, *IRE1α*, and *CHOP*, reverse transcription quantitative real-time polymerase chain reaction (RT-qPCR) was conducted on a LightCycler 480 II system (Roche, Basel, Switzerland) with SYBR Green chemistry. The primer sequences for these genes are detailed in Table 2. 

RT-qPCR reactions were performed at a final volume of 20 µL containing SYBR Green I Master Mix, forward and reverse primers, nuclease-free water, and cDNA template. Amplification was carried out with an initial denaturation at 95 °C for 10 min, followed by 45 cycles of denaturation at 95 °C for 10 s, annealing at 57–59 °C for 10 s depending on the target gene, and extension at 72 °C for 15 s. Melting curve analysis was performed to confirm amplification specificity.

Relative mRNA expression levels were calculated using the 2^−ΔΔCt^ method [40] and normalized to *β-actin* as the housekeeping gene.

All experimental groups initially consisted of six animals (n = 6). For RT-qPCR analysis, one sample from the Control and CMC groups was excluded due to technical reasons during RNA quality assessment and/or amplification procedures. Therefore, RT-qPCR analyses were performed using five samples in the Control and CMC groups (n = 5) and six samples in the remaining groups (n = 6). 

### 4.6. Histopathological Examination

Rats were euthanized under general anesthesia by cervical dislocation, after which kidney tissues were excised and immersed in 10% buffered formalin for fixation. Following fixation, specimens were processed through ascending concentrations of ethanol, cleared with xylene, and subsequently embedded in paraffin wax. Paraffin blocks were sectioned at a thickness of 5 μm using a rotary microtome, and the resulting sections were placed on chromalum gelatin (CAG)-coated microscope slides. Tissue sections were stained with hematoxylin and eosin (H&E) and evaluated microscopically using a Zeiss Primo Star microscope (Zeiss, Oberkochen, Germany) [41]. Histopathological examinations were conducted in a blinded manner. During histopathological scoring, the evaluated parameters were assessed separately for each tissue, and 10 different areas per section were examined in each group to determine lesion frequency and severity. Histopathological lesions were semi-quantitatively scored as absent (−), mild (+), moderate (++), severe (+++), and very severe (++++).

### 4.7. Statistical Analysis

Data processing and statistical evaluations were carried out using Jamovi (version 2.6.44) and JASP (version 0.95.4). Quantitative data are presented as mean ± standard deviation (SD). Distributional assumptions were examined using the Shapiro–Wilk normality test. Variables showing a normal distribution were analyzed by one-way ANOVA, whereas variables that did not satisfy normality assumptions were evaluated using the Kruskal–Wallis test. Pairwise comparisons following significant overall differences were conducted with Tukey’s multiple comparison procedure for parametric datasets and Dunn’s test for non-parametric datasets. Statistical significance was defined as *p* < 0.05.

## 5. Conclusions

The outcomes of the current investigation demonstrate that NAR alleviates MTX-induced renal injury by modulating pathways associated with oxidative stress, inflammation, ER stress, and apoptosis. NAR treatment improved antioxidant defense-related gene expression and reduced inflammatory, ER stress-related, and apoptotic markers. However, the persistence of certain histopathological alterations suggests that structural recovery of renal tissue may require longer time periods than molecular changes. Overall, these findings highlight the potential nephroprotective impacts of NAR against MTX-caused nephrotoxicity.

## Figures and Tables

**Table 1:** Histopathological scoring of renal tissue lesions.

	Mean Scores *						
Renal Tissue Lesions	Control	CMC	NAR100	MTX	MTX+NAR25	MTX+NAR50	MTX+NAR100
Cortical congestion	−	−	−	++	++	+	+
Glomerular atrophy	−	−	−	+	+	+	+
Inflammatory cell infiltration	−	−	−	++	++	+	+
Glomerular lobulation	−	−	−	+	+	+	+
Tubular degeneration		−	−	++	++	++	+
Enlargement of Bowman’s capsule	−	−	−	++	++	+	+

* −: no abnormality; +: mild abnormality; ++: moderate abnormality.

**Table 2:** Primer sequences used for RT-qPCR analysis.

Gene	Forward Primers	Reverse Primers	Accession Number	Product Size (bp)
*CAT*	5′-CTGAGAGAGTGGTACATGCA-3′	5′-AATCGGACGGCAATAGGAGT-3′	NM_012520.2	130
*GPx*	5′-CAAGGTGCTGCTCATTGAGA-3′	5′-ATGTCCGAACTGATTGCACG-3′	NM_030826.4	139
*NF-κB*	5′-AGTCCCGCCCCTTCTAAAAC-3′	5′-CAATGGCCTCTGTGTAGCCC-3′	NM_001276711.1	106
*GRP78*	5′-CATGCAGTTGTGACTGTACCAG-3′	5′-CTCTTATCCAGGCCATATGCAA-3′	NM_013083.2	143
*PERK*	5′-GATGCCGAGAATCATGGGAA-3′	5′-AGATTCGAGAAGGGACTCCA-3′	NM_031599.2	198
*IRE1α*	5′-GCAGTTCCAGTACATTGCCATTG-3′	5′-CAGGTCTCTGTGAACAATGTTGA-3′	NM_001443666.1	163
*ATF-6*	5′-TCAACTCAGCACGTTCCTGA-3′	5′-GACCAGTGACAGGCTTCTCT-3′	NM_001107196.1	130
*CHOP*	5′-GAAGCCTGGTATGAGGATCT-3′	5′-GAACTCTGACTGGAATCTGG-3′	NM_001109986.1	209
*Caspase-12*	5′-CACTGCTGATACAGATGAGG-3′	5′-CCACTCTTGCCTACCTTCC-3′	NM_130422.2	138
*Bax*	5′-TTTCATCCAGGATCGAGCAG-3′	5′-AATCATCCTCTGCAGCTCCA-3′	NM_017059.2	154
*Bcl-2*	5′-GACTTTGCAGAGATGTCCAG -3′	5′-TCAGGTACTCAGTCATCCAC-3′	NM_016993.2	214
*Caspase-8*	5′-GATGAGGCAGACTTTCTGCT-3′	5′-CATAGTTCACGCCAGTCAGGAT-3′	NM_022277.2	163
*Caspase-9*	5′-ACGTGAACTTCTGCCCTTCC-3′	5′-GGTCGTTCTTCACCTCCACC-3′	NM_031632.3	117
*Caspase-3*	5′-ACTGGAATGTCAGCTCGCAA-3′	5′-GCAGTAGTCGCCTCTGAAGA-3′	NM_012922.2	270
*β-actin* (ACTB)	5′-CAGCCTTCCTTCTTGGGTATG-3′	5′-AGCTCAGTAACAGTCCGCCT-3′	NM_031144.3	360

## Data Availability

The original contributions presented in this study are included in the article. Further inquiries can be directed to the corresponding author.
